# Round the Hospitals

**Published:** 1917-03-03

**Authors:** 


### ROUND THE HOSPITALS.

The nursing world have been expecting a declaration of policy on the part of the Manchester Branch of the National Union of Trained Nurses, following the censure on the present executive committee of the Union at the large and representative meeting held on the 10th February. We understand that the resolution of the Manchester Branch is highly approved by a large number of the older members of the Union who are exceedingly dissatisfied with the party which has captured the executive for the moment. Many members are stoutly opposed to the introduction of politics, and resolved to eliminate this element and to restore to the Union the cardinal principle for which it was founded—namely, to maintain the highest ideals of the nursing profession. The executive and its defenders are fond of posing as persons who are fighting to secure "to the rank and file of the nursing profession the right to speak, think, and judge for themselves concerning their own affairs," which is precisely what the Royal College of Nursing is established to secure and will guarantee to British trained nurses. In practice the executive has appointed as secretary a lady who is not a certificated trained nurse, and they sent her as a speaker to a recent meeting of the Bath Branch. She claimed to speak as the representative of the only "nationally organised self-governing professional society of nurses." Could make-believe and bold effrontery on the part of an Executive Committee of an organisation of nurses take any step better calculated to arouse the just wrath of British nurses, or to expose the unfitness of the present executive to be entrusted with the affairs of a self-governing professional society of nurses, whether nationally organised or otherwise? We hope that Lancashire women, with the sterling grit which is always attributed to them, will lead a movement to reorganise and shake themselves free from humbug and pretence, by establishing a Reformed National Union of Trained Nurses which shall be a reality and not a sham. The nurses can so be given guarantees that the R.N.U.T.N. will continuously devote itself to the maintenance of the highest ideals of the nursing profession, and be conducted by nurses for nurses under the guidance and control of members of their profession, in whom they have confidence. Miss E. L. C. Eden, the secretary, who is not a certificated trained nurse, is reported to be going round declaring that the Union, under its present managers, is out to fight the College to the death—that is, to fight for the division of the nursing profession, in the hope of weakening it with the object of securing for the Leader and the Executive Committee their immediate ambitions. We believe the Trained Nurses, Sisters, and Matrons of Great Britain will resent this impertinence, and speedily teach such impotent disturbers to mind their own business.

---

It is our privilege warmly to congratulate the upwards of 470 Matrons, Sisters, and Staff Nurses whom His Majesty the King has honoured by conferring upon each of them the Royal Red Cross as R.R.C. or A.R.R.C. members of this, the highest Order trained nurses can have conferred upon them. On going through the list we are grateful to find that His Majesty who, like Her Majesty the Queen, is ever watchful to mark noble service in the cause of the sick when efficiently rendered, has included in the list of honours a large number of those whose claim to recognition we have often advocated on the ground that they are Homeland

**ROUND THE HOSPITALS** *(continued)*.

Workers in Wartime. This departure is memorable in the history of the Order and of hospital and nursing work in these realms. There can be no doubt, as all who are most familiar with the actual circumstances of work in the Homeland during wartime will agree, despite the disappointment which so many of us feel that age, or claims of pressing and urgent duties in the Homeland, have prevented us from sharing the privilege of fighting for the Empire and the Nation in foreign lands, that success in wartime rests mainly on the efficiency of the organisation at home. That is why we welcome this, the latest *Gazette* which does deserved honour to Homeland workers who are indeed worthy of the Empire and the Nation.

FOR two successive weeks we have dwelt upon the splendid opportunity every matron and lady superintendent of nursing, and indeed every sister, has to help her nurses to help themselves by placing their names upon the Register of the Royal British College of Nursing. Our thanks and recognition are due to an ever-increasing number of matrons and others who have realised their responsibility and duty by thus helping their nurses to help themselves. The latest example comes from a Principal Matron who has probably the largest and most onerous charge in the whole country. She writes: "I have advised all my trained staff, civil and territorial, to get their names registered on the Roll of the College of Nursing without delay. I believe a very large number have already done so, and I feel sure most of my nurses will register. I have done everything I can to urge them not to wait, but to act now." On page 365 of *The Nursing Mirror* of February 10 some striking figures are given showing the really magnificent way in which trained nurses throughout Great Britain are coming forward spontaneously to place their names on the Register and to recruit every nurse whom they know to follow their example. Three thousand such recruits on the College Register by the nurses' own efforts in a little over two months shows conclusively that the British Nursing Profession is solid for the College, and that it is something which British Nurses ardently long for and wholeheartedly believe in and trust.

LAST week, in addressing the Matrons, Lady Superintendents, and Sisters, we intimated that Miss Rundle is prepared to accept invitations sent to her at the College of Nursing, 6 Vere Street, Cavendish Square, W., to come and help the matrons to help the nurses to help themselves. Since our last issue Miss Rundle and Miss Cowlin, secretary and assistant secretary of the College of Nursing, visited the Bethnal Green Military Hospital on February 21 on behalf of the College of Nursing. The nurses displayed great interest in the addresses these two ladies gave, and much intelligence. Their welcome was as warm as it was much appreciated, and the results to the Register will no doubt tend to strengthen it. Many more such meetings, please! In this connection it is a privilege to congratulate Miss Dodds, the matron of the Bethnal Green Military Hospital, on His Majesty's award to her of the R.R.C.

